# Magnetic expression in kerogen reveals impact on fluid transport

**DOI:** 10.5194/mr-3-125-2022

**Published:** 2022-07-29

**Authors:** Benjamin Nicot, Jean-Pierre Korb, Isabelle Jolivet, Hervé Vezin, Didier Gourier, Anne-Laure Rollet

**Affiliations:** 1 TotalEnergies, Avenue Larribau, 64000 Pau, France; 2 Sorbonne-Université, CNRS, PHENIX, 75005 Paris, France​​​​​​​; 3 Univ. Lille, CNRS, UMR8516 – LASIRe 59000, Lille, France; 4 Chimie ParisTech, PSL University, CNRS, Institut de Recherche de Chimie de Paris (IRCP), 75005 Paris, France

## Abstract

How can the transport of fluids in a confined and complex mixed organic/inorganic matrix be far below the expected value from a topological aspect? A good example of this situation is oil shales. Oil and gas shales are source rocks in which organic matter has matured to form hydrocarbons. They exhibit a dual porous network formed by the intertwining of mineral and organic pores that leads to very low permeability. Still, the exact origin of this extremely low permeability remains somehow unclear. The present communication addresses this important question and provides novel insights on the mechanisms that strongly hinder fluid diffusion in such materials. By combining nuclear and electronic magnetic resonance techniques with SEM imaging, we show evidence that magnetic interaction occurs in kerogen. This results from a magnetic coupling between vanadyl present in porphyrins and the organic matrix. We demonstrate that such coupling retards fluid diffusion and is reversible. This key dynamical feature explains the extremely low mobility of oil in shale rocks. This phenomenon may be a more general feature occurring in several systems where fluids are confined in a complex hierarchical matrix that embeds both organic and inorganic radicals resulting from the aging process.

## 1 Introduction

Millions or billions of years ago, the diagenesis of organic-rich sediments led to the formation of rocks consisting of a mixture of minerals and solid carbonaceous matter called kerogen. These more or less hydrogenated carbonaceous materials carry information about ancient life forms and their environments and are key for understanding the various attempts made during life evolution (Derenne et al., 2008). Metabolism of most living systems, even the most primitive ones, is based on various metalloporphyrin complexes. Among the most important metal ions, Mg ${}^{2+}$ and Fe ${}^{2+}$ are involved in anoxygenic and oxygenic photosynthesis and in anaerobic and aerobic respiration, respectively. During post-mortem degradation of the biological matter in the sediment, metal ions of porphyrins are substituted by vanadyl ions VO ${}^{2+}$ , giving very stable vanadyl porphyrins (VOP) (Breit and Wanty, 1991).

These organic-rich rocks are also of crucial economical interest as they are unconventional oil and gas source rocks. Furthermore it requires the use of hydraulic fracturing to compensate for the extremely low mobility of hydrocarbons, yielding adverse environmental consequences (Spellman, 2013).

An accurate petrophysical evaluation of these rocks (volume of resources in place: porosity, hydrocarbon saturation, permeability and fracability) has appeared more challenging in tight organic shales than in conventional reservoirs (Sondergeld et al., 2010; Le Bihan et al., 2014). In particular, the evaluation of water/hydrocarbon saturations (the fraction of porosity filled by water or hydrocarbon) using classical “Dean–Stark” and “retort” techniques has proved to be inaccurate (Handwerger et al., 2011, 2012; Simpson and Fishman​​​​​​​, 2015). Recently, low-field nuclear magnetic resonance (NMR) has solved this issue using two-dimensional $T_{1}$ – $T_{2}$ NMR correlation maps (Nicot et al., 2015). Very high $T_{1}/T_{2}$ ratios have been found experimentally, both on shale samples (Nicot, et al., 2015) and kerogen isolates (Singer et al., 2016). The origin of such a high $T_{1}/T_{2}$ ratio and its dependence on Larmor frequency has been explained using field cycling NMR relaxometry (Korb et al., 2014). We have used a model of relaxation for interpreting the nuclear magnetic relaxation dispersion (NMRD) as well as 2D correlation spectra $T_{1}$ – $T_{2}$ of oil and brine embedded in shale rocks to extract some relevant dynamical parameters. Our modeling captures the main point that the probability of reencounters between the mobile probed molecules and the fixed paramagnetic species is drastically enhanced by the low dimensionality of the local geometry. Other two-dimensional NMR experiments have been also proposed in petroleum industry to relate diffusion and transverse relaxation (Korb et al., 2015). Other geometries of pores could exist in some other shales, but the essential features of the NMR relaxation are fully considered by our modeling, i.e., a liquid that makes numerous back and forward dynamics in the proximity of important sources of relaxation. The numerous experimental techniques used prove the unicity of our modeling procedure. Now remains the issue of hydrocarbon transport and its relationship with the multiscale structure of kerogen.

In the present communication, we aim at addressing three fundamental questions linked to these non-conventional microporous materials. (i) What is the main physical origin of the extremely low permeability of these materials? (ii) How can fluids be localized, either in mineral or organic porosities? (iii) Finally, how can their individual dynamics be characterized?

To address these issues, we combined the use of original multiscale and multidimensional nuclear magnetic relaxation and advanced pulsed electron magnetic resonance (EMR) with imaging techniques. We first describe the multiscale intertwined structure of the shale samples under study. We then focus on the multiscale structure of kerogen, and we observe kerogen swelling in the presence of oil, through a modification of paramagnetic nanostructures. We observe a magnetic phenomenon appearing within the solid kerogen structure as a result of a coupling between vanadyl ions and a carbon radical. Finally, we reveal the nature of both fluids' dynamics locked in this complex porous medium.

## 2 Methods

### 2.1 Samples

The samples studied here are shales coming from the Late Jurassic Vaca Muerta Formation, located in the Neuquén Basin in northern Patagonia, Argentina. This formation hosts major deposits of shale oil and shale gas. Three samples (diameter 10 mm, length 16 mm) were studied and yielded similar results. Samples were studied in different states: the “as received” state, in which the shale contains the native fluids, and the “dry” state, in which the rock does not contain any fluid. The kerogen was then isolated by a HCl/HF acid demineralization process (Durand and Nicaise, 1980). Experiments presented here include experiments on “dry” kerogen isolates and on “dodecane-impregnated” kerogen isolates (after submitting the kerogen isolates to dodecane).

### 2.2 Electron microscopy

To perform elementary microanalyses and nanostructural observations by SEM (scanning electron microscopy) coupled with EDS (energy dispersive X-ray spectrometry), the samples were polished mechanically down to 1/4 µ m with diamond suspensions and then ion-milled with the Fischione 1060 at 5 KV and with an argon gun at tilt angles of 5 and 2 ${}^{\circ }$ . To ensure a good conductivity on the sample surface and maintain a good image quality for SEM observations, the plugs were coated with platinum.

To assess the rock heterogeneity, a quantitative mineralogical map was acquired on a FEI Quanta 650 electron microscope equipped with two energy dispersive X-Ray spectrometers (EDS Bruker X-Flash) and combined with the mineral identification software package Maps-Nanomin. The interpretation method used in the Nanomin software was previously calibrated with quantitative data based on X-Ray diffraction and X-ray fluorescence (Fialips et al., 2018). Additional SEM observations at different length scales were then performed locally to qualitatively identify the different types of porosity in the organic matter and in between the minerals following the classification proposed by Louks et al. (2012).

**Figure 1:**
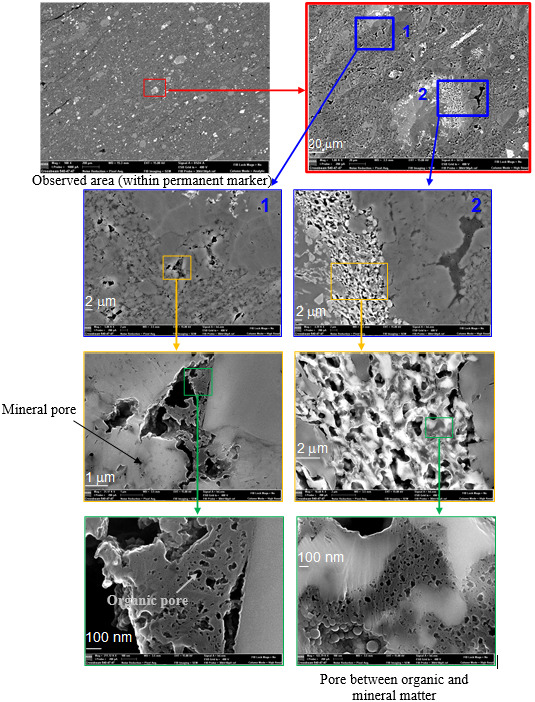
Visualization of the microstructure and the arrangement of mineral grains versus organic matter using SEM imaging at different length scales from 20 µ m to 100 nm (from top to bottom). The successive zoomed-in views focus on the meso- and macroporosity in the organic matter.​​​​​​​

**Figure 2:**
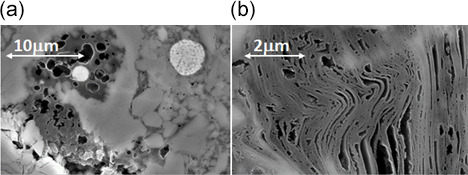
SEM images of shale samples at different resolution. **(a)** A “sponge-like” kerogen porosity and a framboidal pyrite, **(b)** a lamellar clay structure filled by porous kerogen.

**Figure 3:**
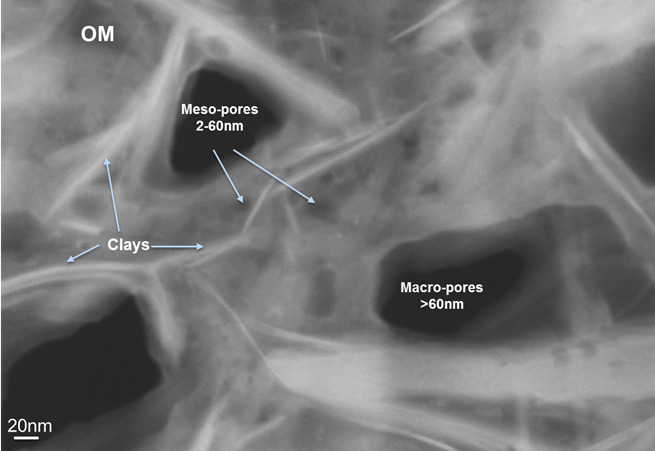
SEM-STEM imaging performed on a mud area enriched in organic matter (OM) and clays. Visualization of macro- and mesoporosity of few nanometers of equivalent diameters, which are either connected or distant of few nanometers.

To quantify the porosity by image processing from the nanometric scale up to the micrometric scale (typically in an equivalent diameter range from 10 nm to a few micrometers), large-area imaging was performed on a Zeiss Crossbeam 540 electron microscope. Backscattered electron images were acquired at low acceleration voltage (5 KV) and a current beam of 100–200 pA to cover a representative area of 480 µ m × 240 µ m at a resolution of 5 nm (pixel size). Segmentation of organic matter and pores was then performed on filtered images using the Ilastik software. The classification of the different types of porosity (mineral vs. organic matter hosted pores), performed using the software Visilog, was then carried out using current image processing based on mathematical morphology, where the pixels describing the pores neighboring the organic matter are detected after a dilation operation. The mineral hosted pores were then deduced by subtraction. From the image, the surface of each pore is extracted, and whatever its shape, a diameter is calculated for an equivalent disk. The pore size distributions for each pore type and the derived fractal dimensions were calculated from this image classification (see Figs. 1–3​​​​​​​).

Further observations at higher resolution were also performed on the Zeiss Crossbeam 540 electron microscope on ultra-thin sections of roughly 100 nm of thickness, using the SEM-STEM (transmitted and scattered electrons detector allowing observations in transmission in a scanning electron microscope with an optimized resolution under the nanometer). The main interest of using this technique is to complete the SEM imaging to go further in the qualitative description at higher magnification of the porous network in the organic matter (Fig. 3).

### 2.3 NMR relaxation measurements

Two-dimensional $T_{1}$ – $T_{2}$ maps were acquired at 2.5 and 23 MHz on Oxford Instrument spectrometers with an inter-echo time TE = 200 µ s and inversion recovery varying from 70 µ s to 1 s in 200 values. The results were processed using a 2D inverse Laplace transform (Venkataramanan et al., 2002). The temperature of the samples was 21 ± 1 ${}^{\circ }$ C. Measurements were performed at 2.5 MHz on cylindrical samples of 30 mm diameter and 50 mm height, while measurements at 23 MHz measurements were performed on samples of 10 mm in diameter and 15 mm height.

### 2.4 Fast-field cycling NMR relaxometry

Multi-frequency NMR relaxation dispersion of longitudinal relaxation rate (NMRD) was performed on a fast-field cycling spectrometer from Stelar s.r.l., Mede, Italy. The main interest of using this NMR technique is to explore a large magnetic field range, allowing us to sense a large range of fluctuations to which the nuclear spin relaxation is sensitive in confinement. The measurements were performed on samples 9 mm in diameter and 15 mm in height. At each magnetic field associated with a ${}^{1}$ H Larmor frequency, varying from 10 kHz to 35 MHz, a measurement of the longitudinal relaxation time $T_{1}$ is performed and processed using an in-house 1D inverse Laplace transform, leading to a bimodal $T_{1}$ distribution, which allows the respective oil and brine contributions to be separated.

### 2.5 Electron magnetic resonance

The measurements were performed on samples of 8 mm in diameter and 15 mm in height for continuous-wave (CW) experiments and a piece of 3 × 3 mm dimension for pulsed EMR experiments. Continuous-wave EMR spectra (CW-EMR) were recorded at the X-band ( ≈ 9.4 GHz) at room temperature using a Bruker ELEXSYS E500 spectrometer equipped with a 4122SHQE/011 resonator. Pulsed EMR experiments were carried out at 5 K with a Bruker ELEXSYS E580 X-band spectrometer equipped with a Bruker cryostat “cryogen-free” system.

HYperfine Sublevel CORrElation (HYSCORE) spectroscopy (Hofer, 1994) was used to reveal hyperfine interactions of the electron spins of carbonaceous matter with ${}^{13}$ C ( I = 1/2 ; 1.1 % abundance), ${}^{1}$ H ( I = 1/2 ; 100 % abundance) and ${}^{29}$ Si ( I = 1/2 ; 4 % abundance) nuclei. In this technique, a spin echo is generated by the pulse sequence π/2 – τ – π/2 – $t_{1}$ – π – $t_{2}$ – π/2 – τ –*echo*. The angles π/2 and π represent the flip angles of the electron magnetization. Its intensity is measured by varying the times $t_{1}$ and $t_{2}$ at constant time τ in a stepwise manner. The lengths of the π/2 and π pulses were fixed at 16 and 32 ns, respectively. A total of 256 × 256 data points were collected for both $t_{1}$ and $t_{2}$ at increments of 20 ns. The τ value was set at 136 ns for all the samples. The unmodulated part of the echo was removed using second-order polynomial background subtraction. The magnitude spectrum was obtained after 2D Fourier transformation of the spectra using a Hamming apodization function.

For the Pulsed ELectron DOuble Resonance (PELDOR) experiments, a four-pulse sequence with a Gaussian, non-selective observer and pump pulses of 8 or 16 ns length with 280 MHz frequency separation was used. An eight-step phase cycling was performed together with 0– π phase cycling to remove unwanted effects of running echoes from the DEER trace. The evaluation of the DEER data was performed using DeerAnalysis2018 (Jeschke et al., 2006). The background of the primary DEER traces was corrected using exponential functions with homogeneous dimensions. A model-free Tikhonov regularization was used to extract distance distributions from the background corrected form factors (Jeschke et al., 2002).

## 3 Results

### 3.1 Multiscale intertwined structure of organic and inorganic porosities

In general, shales are fine-grained and laminated sedimentary rocks consisting in a mixture of several minerals (clays, quartz, calcite, feldspars, pyrite, etc.) and solid organic matter (kerogen) and pores. These pores can be located in the mineral phase and in the organic matter and can contain either hydrocarbons or water. Understanding fluid transport in such complex structures requires the knowledge of their hierarchical organization from nanometer to micrometer scales and of the intertwined nature of the porous network.

Scanning electron microscopy was performed on the samples in order to reveal their microstructure. Making successive zoomed-in views (Fig. 1) allows the structure of the sample to be studied at various scales, taking into account samples' heterogeneity. The rock is composed of pure minerals grains and a nanotextured mud formed by a mixture of clays, micro quartz, calcite debris and organic matter.

Several different types of porosity can be identified at the micrometric scale. The two main types observed in organic matter are the following (all porosity types are described in the Supplement): a sponge-like organic matter (Fig. 2a) and a lamellar organic porosity, intricated in the clay structure (Fig. 2b).

Interconnected pores networks rather appear to be present in the nanostructured mud areas. Further investigations were carried out by SEM-STEM in these mud areas to study the nanostructure of the porous network, as shown in Fig. 3. Typically, we show evidence of a tight network of mesopores and macropores that can be connected by nanopore throats a few nanometers away from each other.

Advanced image processing of zoomed-in SEM images allows for thresholding of the image to distinguish organic matter, organic porosity (which represents 75 % to 85 % of the porosity visible on SEM) and mineral porosity. The relation between the number of pores N(R) and pore size R is a power law $N(R)∼R^{-D_{f}}$ , where $D_{f}$ is the surface fractal dimension. A fractal dimension of about 2.3 was found for the three samples tested, in agreement with Curtis et al. (2010). Here the surface fractal dimension characterizes the self-similarity of the pore geometry between the lower (2.5 nm) and upper (630 nm) boundaries. This continuous distribution shows that the solvent accessible surface area $S(R)∼R^{2-D_{f}}$ becomes very large for the dominant contribution of the small pore sizes. This is of particular importance for the interpretation of the NMRD data, especially at low Larmor frequency, where the longitudinal relaxation rates $1/T_{1}$ , that is proportional to the specific surface area $S_{P,\mathrm{NMR}}$ , become very large.

This provides a quantitative measurement of the hierarchical character of the porous microstructure already shown in Fig. 1. Therefore, electronic microscopy reveals the hierarchical structure of kerogen, with porosity appearing as a sponge-like fractal, either in patches or filling the lamellar structure of clay minerals.

**Figure 4:**
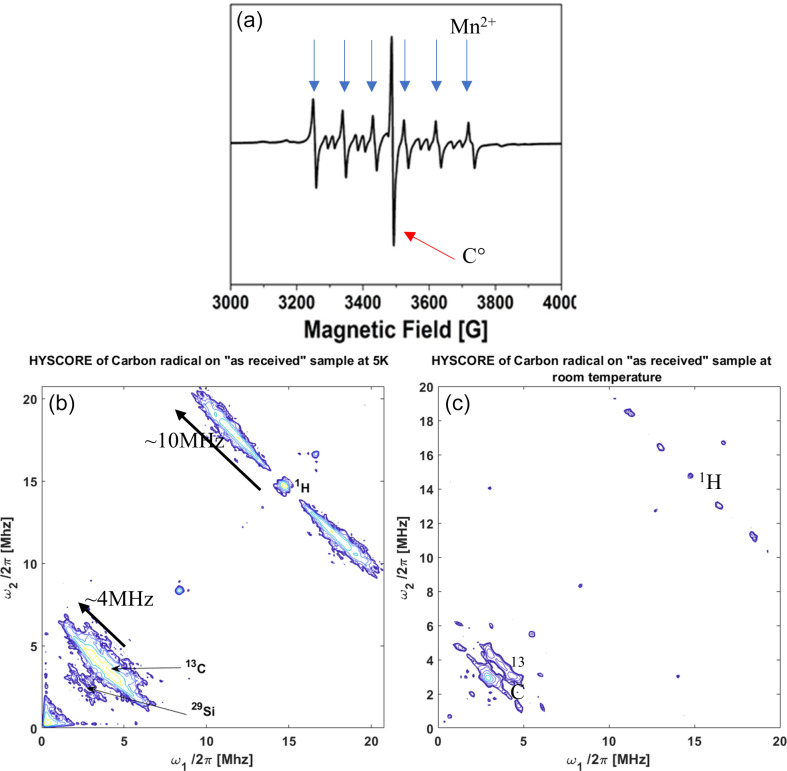
EMR experiments performed on the rock sample in the “as received” state. **(a)** Continuous-wave EMR spectrum performed at room temperature; electron paramagnetic resonance (EPR) lines are indicated by blue arrows for Mn ${}^{2+}$ and a red arrow for the carbon radical C ${}^{⚫}$ . **(b)** Two-dimensional EMR HYSCORE of carbon-centered radical C ${}^{⚫}$ recorded at 5 K; the inset shows the echo detected EMR signal. **(c)** Two-dimensional EMR HYSCORE of C ${}^{⚫}$ recorded at room temperature.

Since clays and kerogen are well known for containing paramagnetic species, the structure of such complex materials can also be investigated by quantitative EMR. Continuous-wave (CW) EMR spectrum (Fig. 4a) unambiguously reveals the presence of paramagnetic Mn ${}^{2+}$ ions (six hyperfine lines) and an organic radical labeled C ${}^{⚫}$ (intense single line centered at g = 2). By integrating these calibrated spectra, we found ≈ 4.50 × 10 ${}^{19}$ Mn ${}^{2+}$ and ≈ 1.20 × 10 ${}^{17}$ carbon radicals (C ${}^{⚫}$ ) per gram of rock. Moreover, the continuous-wave spectrum of extracted kerogen (shown in the Supplement) only shows the single line of carbon radicals around g = 2. This proves that the carbon radicals are located in kerogen while manganese impurities belong to the mineral phase.

Two-dimensional HYSCORE (HYperfine Sublevel CORrElation) spectroscopy (Hofer, 1994) experiments performed on “as received” shale samples (Fig. 4b) reveal the local nuclear environment of C ${}^{⚫}$ radicals ( ${}^{13}$ C at 3.7 MHz and ${}^{1}$ H at 14.5 MHz). The nature of the neighboring coupled nuclei (their Larmor frequency) is measured along the first diagonal, and the strength of their couplings is measured along the anti-diagonal (4 and 10 MHz for ${}^{13}$ C and ${}^{1}$ H, respectively). Such values are typical of what can be measured in primitive organic matter (Gourier et al., 2008).

As will be demonstrated below, the observed ${}^{1}$ H signal mainly arises from the interaction between the kerogen radical C ${}^{⚫}$ and hydrogen atoms of trapped oil. The fact that such interaction is visible in the 2D spectrum at 5 K requires a quasi-static environment of the protons around the radical. Moreover, the ${}^{1}$ H pattern intensity decreases when recorded at room temperature but does not fully vanish (Fig. 4c). This points to a very low mobility of the oil protons as a hyperfine coupling of ≈ 10 MHz comparable with that found at 5 K can still be observed at room temperature.

Once the paramagnetic species have been identified and quantified, we address the question of the uniformity of their spatial distribution. EMR spatial and spectral/spatial (C ${}^{⚫}$ or Mn ${}^{2+}$ ) imaging (shown in the Supplement) reveals a homogeneous distribution of paramagnetic species and a nearly constant C ${}^{⚫}$ / Mn ratio at a spatial resolution of 1 µ m. This proves that despite the extreme heterogeneity of the sample, the sources of NMR relaxation (paramagnetic impurities) are homogeneously distributed within the sample.

### 3.2 Interactions between oil and kerogen

Having identified the two types of paramagnetic species (Mn ${}^{2+}$ in minerals and C ${}^{⚫}$ radicals in kerogen), we used them to probe the relations between the three components of the shale: minerals, kerogen and oil. Considering the intertwined structure of clays and kerogen evidenced by electronic microscopy, we performed EMR experiments to probe the Mn ${}^{2+}$ –C ${}^{⚫}$ interactions between clays and kerogen.

**Figure 5:**
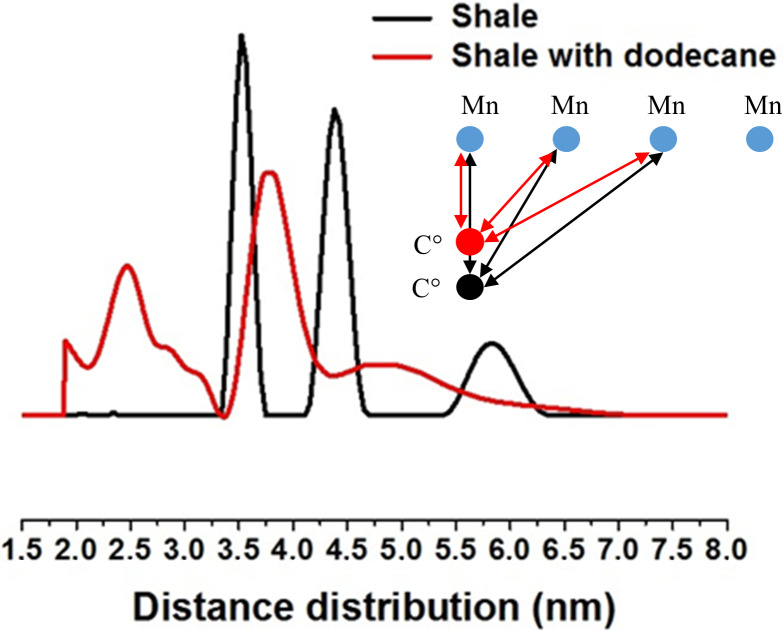
Distribution of carbon (C ${}^{⚫}$ )–manganese distances in the the dry shale sample (black) and after spontaneous imbibition of dodecane (red), measured at 40 K using the four-pulse PELDOR sequence. All the distances measured are consistent with the schematic nanostructure of paramagnetic species proposed in the inset.

We performed Pulsed ELectron DOuble Resonance (PELDOR) experiments (Jeschke et al., 2006), which allows the distances between paramagnetic centers to be calculated by refocusing the dipolar interaction between two paramagnetic centers, namely Mn ${}^{2+}$ in the mineral phase and C ${}^{⚫}$ in solid kerogen. Figure 5 shows the distribution of Mn ${}^{2+}$ –C ${}^{⚫}$ distances obtained in dry shale and in the shale impregnated with dodecane. Three sets of distance distributions centered on 3.5, 4.5 and 6.0 nm are measured in the dry shale, while adding dodecane to the shale results in a significant shortening of these distances (broad distribution around 2.5, 3.7 and 4.8 nm). The striking point is that the structure is conserved upon swelling. Indeed, these distances (with or without dodecane) are consistent and provide an estimate of the mean Mn–Mn distance of about 2 nm. It is worth pointing out that a similar result can be obtained by estimating the Mn–Mn distance from the overall Mn concentration ( $\eta_{S}$ = 4.5 × 10 ${}^{19}$ Mn per gram of rock), the rock density ( $\rho_{S}$ ≈ 2.6 g cm ${}^{-3}$ ) assuming a uniform spatial distribution.

This PELDOR experiment reveals that dodecane penetrates the nanostructure of solid kerogen inducing swelling, decreasing the distance and therefore increasing the dipolar interaction between a C ${}^{⚫}$ radical in the kerogen and Mn ${}^{2+}$ in the mineral phase. Although swelling has been previously observed at the macroscopic scale (Ertas et al., 2006), this is the first time that swelling is experimentally observed and quantified at the nanometric scale. Moreover, we verified that this phenomenon is reversible: after drying out all the dodecane, the 2D HYSCORE spectrum is the same as the spectrum obtained before dodecane impregnation.

### 3.3 Magnetic interactions of oil inside solid kerogen

In order to probe potential interactions between oil and kerogen, experiments have been performed on dry kerogen and on kerogen impregnated by dodecane.

**Figure 6:**
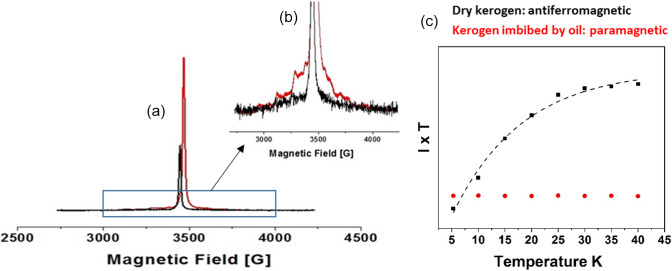
**(a)** Pulses echo field sweep experiments of dry extracted kerogen (black) and kerogen impregnated with dodecane (red) recorded at 5 K. **(b)** Zoomed-in view of the vanadyl porphyrin moiety. **(c)** I × T plot of C ${}^{⚫}$ intensity I versus temperature T between 5 and 40 K for the extracted kerogen (in black) and for the kerogen impregnated with dodecane (in red).

Figure 6a displays the echo field sweep EMR spectra of extracted kerogen, exhibiting a typical single line of 5 G​​​​​​​ linewidth characteristic of C ${}^{⚫}$ radical. In the presence of dodecane impregnation, one observes a drastic increase of the C ${}^{⚫}$ signal and the appearance of a new signal of vanadyl VO ${}^{2+}$ ions (Fig. 6b).

The variation of intensity of the C ${}^{⚫}$ signal as a function of temperature in a range from 40 to 5 K (Fig. 6c) unambiguously characterizes a weak antiferromagnetic behavior of the pure extracted kerogen. This indicates the presence of pairs of radicals with S=1/2 interacting by a weak exchange interaction, giving two states with total spin S = 0 and 1. The state S = 0 is at lower energy, with a J value of - 0.2 cm ${}^{-1}$ . On the other hand, after kerogen swelling with oil, the C ${}^{⚫}$ radical displays a Curie-type paramagnetism); i.e its intensity increases as 1/T with decreasing temperature. The simultaneous increase of C ${}^{⚫}$ and the appearance of VO ${}^{2+}$ signal can be explained by a structural ordering (antiferromagnetic interaction) between VO ${}^{2+}$ and C ${}^{⚫}$ of the kerogen, both having S = 1/2 . The presence of oil induces kerogen swelling that breaks the antiferromagnetic interaction between VO ${}^{2+}$ and C ${}^{⚫}$ radicals, resulting in a pure Curie behavior of C ${}^{⚫}$ radicals. Analysis of the VO ${}^{2+}$ signal by 2D HYSCORE reveals a nitrogen pattern typical of vanadyl porphyrins, an ubiquitous paramagnetic complex of bitumen and oil (Gourier et al., 2010; Ben Tayeb et al., 2015, 2017).

In order to bring to light the interaction between C ${}^{⚫}$ radicals of kerogen and nuclei of dodecane, we investigated their magnetic couplings using 2D-HYSCORE experiments in the dry state of kerogen compared with the dodecane impregnated state.

**Figure 7:**
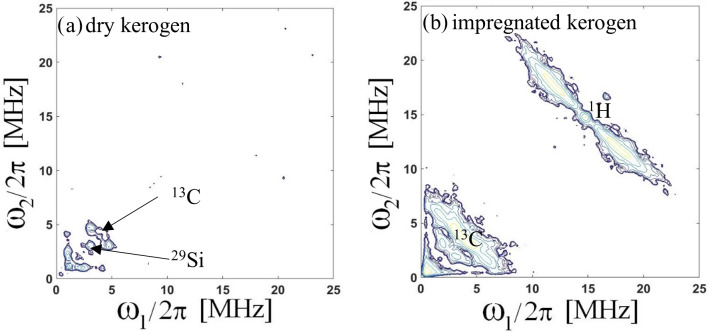
**(a)** Two-dimensional HYSCORE of carbon-centered radicals of extracted kerogen and **(b)** extracted kerogen impregnated with dodecane model oil. All spectra are recorded at 5 K.

For dry extracted kerogen (Fig. 7a), only a weak signal with small couplings with ${}^{13}$ C (at 3.7 MHz) and ${}^{29}$ Si (at 2.9 MHz) is observed. The absence of proton coupling indicates the H / C ratio is low and that the closest protons are at least 5 Å away from the carbon radical. This indicates a high level of maturity of the organic matter (Gourier et al., 2010; Ben Tayeb, et al., 2015, 2017). The presence of silicon can be explained as a mineral residue of the acid attack (demineralization) necessary for extracting kerogen.

After oil impregnation (Fig. 7b), the spectrum exhibits typical proton and carbon patterns, quite similar to the pattern obtained on the “as received” shale (Fig. 4). This implies that the protons and carbons nuclei interacting with C ${}^{⚫}$ belong to the dodecane molecules. Therefore this experiment evidences the very close proximity of the dodecane molecule to the carbon radicals in the heart of the kerogen. This proves that oil molecules are located within organic pores in the solid kerogen. The similarity of the 2D-HYSCORE spectra of dodecane impregnated kerogen (Fig. 7b) and of the pristine shale (Fig. 4a) demonstrates that in the latter, the oil molecules are located in the pores of the kerogen component of the shale. This interpretation is also confirmed by the absence of carbon or proton features in a HYSCORE experiment performed on Mn ${}^{2+}$ transition (Supplement).

After oil removal by evaporation under vacuum, the proton and carbon patterns disappear (Supplement). The structure of the kerogen network is therefore reversible.

All these EMR experiments provide a comprehensive image of all the interactions that are present in the organic part of a shale:

- Two paramagnetic centers are identified in kerogen: a carbon radical C ${}^{⚫}$ and a Vanadyl porphyrin.
- In a dry kerogen, the C ${}^{⚫}$ radical and the Vanadyl porphyrin are coupled in an antiferromagnetic state.
- Oil impregnation breaks this antiferromagnetic coupling.
- Oil molecules are in very close vicinity of the carbon radical and therefore located in the pores of the kerogen.

All these features imply chemical interactions responsible for the magnetic interactions, between the carbon radical and the Vanadyl porphyrin. This interaction is broken when an organic molecule (dodecane in this case) is inserted and replaced by interactions between the radicals and protons on the inserted molecule.

The identification of the type of paramagnetic centers and their quantity is also key for the interpretation of the fluid dynamics investigated by nuclear magnetic relaxation dispersion (NMRD) experiments described in the following section.

### 3.4 Evidence of hindered fluid dynamics

**Figure 8:**
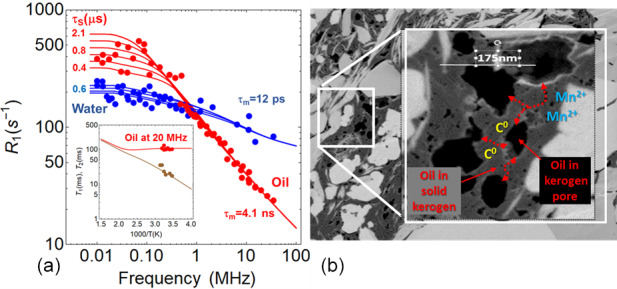
**(a)** NMRD profiles of liquids obtained on an “as received” shale (oil in red, water in blue), representing the variation of the longitudinal relaxation rate $1/T_{1}$ versus the frequency. The inset shows the temperature dependence of relaxation times $T_{1}$ (red) and $T_{2}$ (brown) at 20 MHz for oil obtained on another “as received” shale. The continuous lines are the best fits obtained with the model described in the Supplement, with $\tau_{m}$ and $\tau_{s}$ the translational and surface residence times, respectively. **(b)** Representation of the diffusion–relaxation modeling of oil in the connected pores of kerogen.

In order to assess the physical impact of this magnetic interaction, it is important to accurately probe the dynamics of the liquids (oil and water) in situ and non-invasively. The NMR fast-field cycling technique is perfectly suitable for this purpose (Kimmich, 1997). It explores a large range of Larmor frequency $\omega_{0}/2\pi$ and correspondingly senses longer correlation times of the dipolar fluctuations that are induced by liquid dynamics at the origin of the nuclear magnetic relaxation dispersion (NMRD) of the longitudinal spin-relaxation $R_{1}(\omega_{0})$ (Fig. 8a). Figure 8a is key in this paper. Using high-resolution NMR, we first show evidence the presence of separated oil and water peaks in an “as received” shale. Here, the experimental filled points (red and blue) have been obtained by a Laplace inversion of the longitudinal magnetization decay of an “as received” shale for every Larmor frequency. We observed a net bimodal distribution of $T_{1}$ . The analysis of the apparition/dispersion of these peaks using different procedures of cleaning the sample has shown that the red points belong to the oil and the blue ones to water. In the legends of Fig. 8a, we give the dynamical parameters found for these two fluids with the proposed theory (continuous lines). In the inset, we have analyzed the temperature dependence of the longitudinal $T_{1}$ and transverse $T_{1}$ relaxation times observed at 20 MHz. Here again, the continuous lines represent the best fits obtained with our theory.

Analyzing the NMRD profiles thus allows the surface dynamics of water and oil in micropores to be separated directly. Figure 8a displays the very different observed NMRD profiles associated with these two fluids embedded in an “as received” shale. It represents the longitudinal relaxation rate $R_{1}=1/T_{1}$ versus the Larmor frequency. In a previous paper, we succeeded in identifying the NMRD profiles of these two fluids that cross each other around 1 MHz (Korb et al., 2014). The brine (blue continuous line) exhibits a quasi-logarithmic frequency dependence, whereas oil (red continuous line) follows an inverse square-root behavior with a leveling off at low frequency (details in the Supplement).

In order to interpret these profiles unambiguously, it is crucial to use a relevant theoretical nuclear spin-relaxation model. Here, we just outline the main two features of the used relaxation model described in the Supplement. First, we have observed a mono-exponential decay for each fluid, proving a biphasic fast exchange between proton populations at the pore surfaces and in the bulk. Second, the translational diffusion of both fluids modulates the heteronuclear dipole-dipole interaction between the mobile proton species (water or oil) and the different paramagnetic species fixed at pore surfaces.

The NMRD profile for brine reveals an NMR relaxation induced by a two-dimensional diffusion in the vicinity of Mn ${}^{2+}$ ( S = 5/2 ) at surfaces of lamellar clay mineral (see Fig. 8b) (Korb et al., 2018). All the parameters extracted from our theory were found from the best fits of our NMRD data. Of course some parameters are well known, such as the molecular size δ , and the different densities ρ .​​​​​​​ The specific surface area $S_{P,\mathrm{NMR}}$ is found from the fits and corresponds with the results found in the literature (case of clays). Basically, the typical form of the NMRD profiles allows two correlation times to be found: the surface translational correlation time $\tau_{m}$ of the liquid and $\tau_{s}$ the time of residence of the liquid molecule at the pore surface (Korb et al., 2009). The translational diffusion coefficients are given by the Einstein relations: $D_{\mathrm{surf}}=\delta^{2}/4\tau_{m}$ .

This yields an estimate of the water translational diffusion coefficient at the mineral clay-like surface $D_{\mathrm{surf}}$ = 1.9 × 10 ${}^{-5}$ cm ${}^{2}$ s ${}^{-1}$ for a specific surface area of clay $S_{p}$ = 47 m ${}^{2}$ g ${}^{-1}$ . This local diffusion coefficient is similar to the one of bulk brine and shows that at the local level, the dynamics of water molecules is not hindered (Mills and Lobo, 1989).

On the other hand, the particular frequency dependence observed for oil strongly suggests a relaxation process induced by a highly confined translational diffusion. The model used for interpreting the oil NMRD profiles relies on a quasi-1D translational diffusion of oil at the vicinity of paramagnetic sources of relaxation. We used the nature and concentration of paramagnetic centers (C ${}^{⚫}$ and VO ${}^{2+}$ ) as well as the total spin states S = 1 for the C ${}^{⚫}$ –VO ${}^{2+}$ pairs determined by EMR.

With such inputs, the model is able to fit experimental results quite well, giving information on the dynamical parameters: (i) a specific surface area $S_{p}$ = 233 m ${}^{2}$ g ${}^{-1}$ , (ii) a translational diffusion correlation time $\tau_{m}$ = 4.1 ns, (iii) a relevant kerogen pore size ( R = 0.3 nm) at the maximum of the pore size distribution N(R) and (iv) a very slow translational diffusion coefficient of oil of $D_{\mathrm{surf}}$ = 2.6 × 10 ${}^{-7}$ cm ${}^{2}$ s ${}^{-1}$ . This value of the surface diffusion of oil is representative of the whole pore size distribution. As the pore surfaces are chemically equivalent for the whole pore size distribution that is very large, there is no reason why there will be differences in the translational diffusion at pore surfaces.

To assess the reliability of the NMRD data analysis, we made two supplementary verifications of the proposed model. First, the temperature dependencies of the longitudinal $T_{1}$ and transverse $T_{2}$ relaxation times of oil at 20 MHz for a second sample are displayed in the inset of Fig. 8. As detailed in the Supplement, the asymptotic theoretical temperature dependencies of these relaxation times behave as $T_{1}\propto \surd (\omega_{I}/\tau_{m})=C^{\mathrm{te}}$ and $T_{2}(T)\propto 1/\surd (\tau_{m}\tau_{s}(T))$ , as is observed in the experimental data. Here $\tau_{m}$ and $\tau_{s}$ are the translational and surface residence times, respectively. Second, 2D spin correlation maps $T_{1}$ – $T_{2}$ for oil and brine embedded in shale oils rocks at 2.5 and 23 MHz are displayed in the Supplement. In the same figure, the theoretical evolution calculated at these Larmor frequencies for $\tau_{m}$ = 4.1 ns with 0.4 µ s < $\tau_{s}$ < 2.1 µ s for oil and $\tau_{m}$ = 12 ps with 0.4 µ ss < $\tau_{s}$ < 0.6 µ ss for brine is superimposed and shows quite a satisfactory agreement. The surface translational correlation time $\tau_{m}$ of the liquid and the time of residence $\tau_{s}$ of the liquid molecule at the pore surfaces (Korb et al., 2009) are intrinsically considered in Eqs. (5) and (6) of SEM​​​​​​​ from the basic features of the NMR relaxation model. These 2D NMR measurements are universally used, allowing for a fluid-type downhole in petroleum wells. One should add that pulsed fiend gradient (PFG) NMR also allows for the measurement of self-diffusion in bulk (not at pore surface) when the translational diffusion is the unique process responsible for the dephasing of spins. This supposes that there is no influence of the relaxation. This is not the case in shales due to the large contribution of the paramagnetic species.

**Figure 9:**
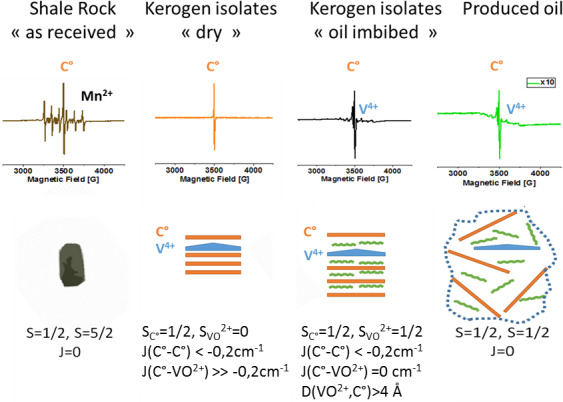
Summary of the different situations encountered in the studied samples, with CW EMR spectra at room temperature (top), scheme of magnetic interactions and related structures (middle), and spin states values and exchange interaction parameters (bottom). Estimated distances between vanadyl and carbon radical d $\left({\mathrm{VO}}^{2+},C^{⚫}\right)$ ​​​​​​​ are also given. In the “as received” shale sample, only two paramagnetic species can be observed (carbon radical C ${}^{⚫}$ and manganese Mn ${}^{2+}$ ), and in the dry kerogen isolate, only the carbon radical signal is observed, whereas in the dodecane-imbibed kerogen isolates, the appearance of a vanadyl signal is observed. These two results demonstrate that the C ${}^{⚫}$ and VO ${}^{2+}$ are antiferromagnetically coupled. This interaction is broken by oil intercalation. The key of the magnetic interaction arises from the vanadyl porphyrin complexes which are coupled with the organic matter. Finally, the analysis of the crude oil produced from this shale shows the presence of small amounts of both organic radicals and vanadyl.

Moreover, low-frequency data in Fig. 8a are rather dispersed, which can be due to different values of the activation energy associated with the time of residence $\tau_{s}$ ranging between 0.4 and 2.1 µ s. This observation is consistent with the simulation of Lee et al. (2016), who consider a wide distribution of residence times for oil in kerogen nanostructure that inhibits the activated desorption of this fluid. The scattering of the $\tau_{s}$ values at low frequency comes from the asymptotic theoretical expression of Eq. (6) of SEM for $\frac{1}{T_{1(\omega_{I})}}\propto \sqrt{\tau_{m}\tau_{s}(T)}$ , which is constant in frequency but dependent on τs , which is dependent on temperature. For an activated process, one has $\tau_{s}\propto \mathrm{exp}(E_{s}/RT)$ , where $E_{s}$ is the activation energy of the surface interaction. The scattering of $\tau_{s}$ thus reveals a scattering of activation energy of the surface interaction as described by Lee et al. (2016). At high frequency, $\frac{1}{T_{1(\omega_{I})}}\propto \sqrt{\frac{\tau_{m}}{\omega_{I}}}$ is independent of $\tau_{s}$ , with a total absence of scattering. This gives a supplementary verification of the proposed theory.

Finally, a very fast penetration of oil within the kerogen has been observed (Nicot et al., 2015). This proves that the different kerogen patches are well connected to each other (Fig. 8b). However, due to the magnetic interactions occurring in kerogen, oil transfers by diffusion very slowly between large organic pores through solid kerogen.

## 4 Discussion and conclusion

The main results can be summarized as follows. The complex hierarchical structure of the shale porous network has been revealed by electronic microscopy and shows the dominant contribution of nanopores. The diffusive nature of both water and oil motion in shales has been proved by NMRD experiments, showing the extreme confinement of oil in kerogen. Kerogen swelling at the nanoscale has been evidenced by EMR spectroscopy performed on kerogen isolates. A magnetic interaction has been discovered, whereby hydrocarbon molecules are locked between carbon radicals C ${}^{⚫}$ and vanadyl porphyrin ions. The reversibility of this magnetic interaction has been evidenced by the appearance and disappearance of a vanadyl signal when dodecane is imbibed in the rock or dried out. These findings are summarized in Fig. 9.

However, vanadyl ions are only observed on the kerogen isolates, never on a shale, even if the swelling is observed (Fig. 5). Therefore, it seems relevant to think that kerogen swelling is spatially limited in the rock due to the presence of minerals, preventing the inhibition of magnetic interaction.

Moreover, these results reflect the high degree of structuration of kerogen, with alternating stacks of kerogen (containing C ${}^{⚫}$ radicals) and vanadyl porphyrins, as sketched schematically in Fig. 9. The magnetic interaction between these stacks could play the role of a real magnetic locking, prohibiting the collective diffusion of oil and therefore preventing long distance fluid transport. This key dynamical feature explains the extremely low mobility of oil in shale rocks.

Finally, these results and hypotheses are strongly supported by the fact that the EMR spectrum of the bulk extracted oil reveals the presence of vanadyl porphyrin, indicating that the fracking method extraction is sufficiently powerful to break this magnetic interaction and release a fraction of vanadyl content in the extracted oil.

The reversible magnetic interaction revealed here by joint NMR and EMR techniques in shales might be a more general phenomenon occurring not only in geological system where organic matter is degraded in confined rocks, but also in various systems where aging processes result in the formation of organic and inorganic radicals. Therefore, the proposed approach could open new areas in various fields where the aging of organic matter is of key interest, such as the food industry (aging over weeks), archeological objects (aging over hundreds or thousands of years) (Binet et al., 2002), and nuclear waste storage (aging over millions of years).

## Supplement

10.5194/mr-3-125-2022-supplement The supplement related to this article is available online at: https://doi.org/10.5194/mr-3-125-2022-supplement.

## Data Availability

all this work has been done by TotalEnergies and therefore the data are not considered public.
