# Supplementary material for: Magnetic expression in kerogen reveals impact on fluid transport
Source: Magn Reson (Gott). 2022 Jul 29;3(2):125–36. doi: 10.5194/mr-3-125-2022 (PMC10539839; doi:10.5194/mr-3-125-2022)
Supplement: The supplement related to this article is available online at: https://doi.org/10.5194/mr-3-125-2022-supplement. [file mr-3-125-supplement.pdf]

Supplement of Magn. Reson., 3, 125–136, 2022
https://doi.org/10.5194/mr-3-125-2022-supplement
© Author(s) 2022. CC BY 4.0 License.

Supplement of

Magnetic expression in kerogen reveals impact on fluid transport

Benjamin Nicot et al.

Correspondence to: Benjamin Nicot (benjamin.nicot@totalenergies.com) and Hervé Vezin (herve.vezin@univ-lille.fr)

The copyright of individual parts of the supplement might differ from the article licence.

Scanning electron microscope results

Scanning electron microscopy was performed on the samples in order to reveal their microstructure. Several different types of porosity can be identified at the micrometric scale and are presented in Figure S1.

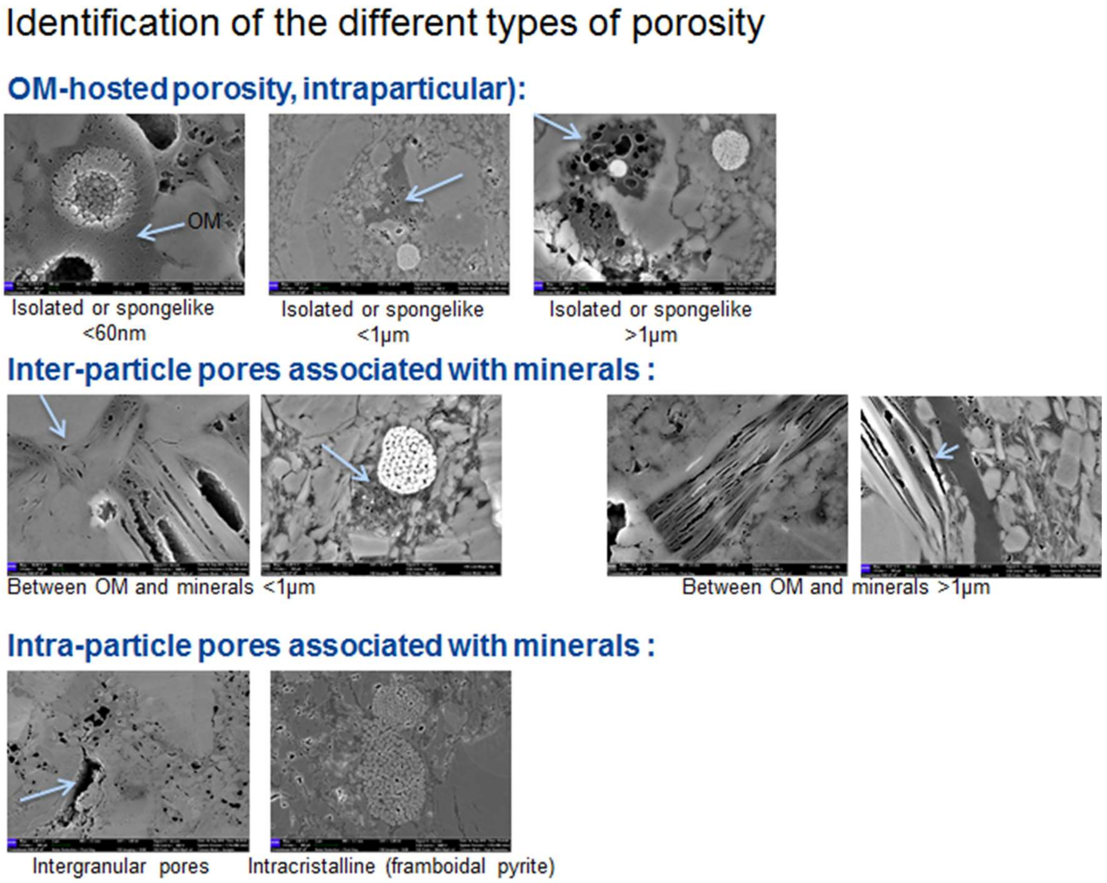

Figure S1 Different types of porosity observed by scanning electron microscope on the studied samples, and classified according

To assess rock heterogeneity and localize region of interest for EDS local analyses, a quantitative mineralogical map was acquired on the FEI FEG-SEM Quanta 650 equipped with 2 Energy Dispersive X-Ray spectrometers (EDS Bruker X-Flash) and combined with the mineral identification software package Maps-Nanomin. The interpretation method used in the Nanomin software was previously calibrated with quantitative data based on X-Ray diffraction and X-ray Fluorescence (Fialips, et al., 2018). The mineralogical composition of these samples, evidenced by EDS (Figure S2) shows the presence of clays (from 15 to 35%, mainly illite/smectite localized in the mud), carbonate (from 10 to 35%,

mainly calcite cementation and micro-particles, containing few amount of manganese), quartz (roughly 30%, micro-particles and grains), feldspars (essentially albite, around 10-15%) and the presence of pyrite (3 to 8%), some of the pyrite being present as framboids.

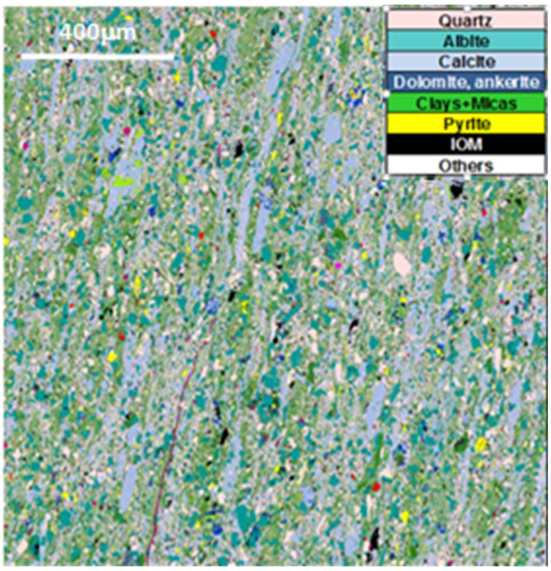

Figure S2: Quantitative mineralogical mapping obtained via SEM-EDS and Maps Nanomin package

An advanced image processing of the SEM images allows the segmentation of the image between organic matter, organic porosity (which represents 75 to 85% of the porosity visible on SEM) and mineral porosity (Figure S3). The pore size distribution of organic porosity reveals a fractal dimension (exponent of the power law relating the numbers of pores to the pore size N(R)) of about 2.3 (from 2.2 to 2.5 on the 3 samples), coherent with what was found by Curtis et al (Curtis, et al., 2010).

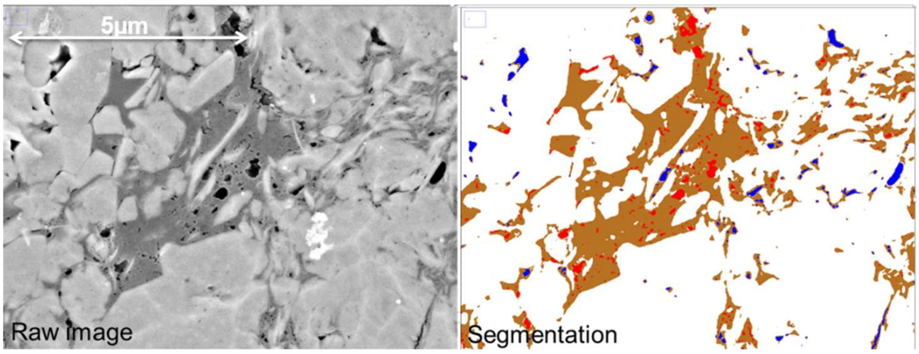

Figure S3: from left to right: Scanning electron microscope image; results of the segmentation to identify kerogen (brown) organic pores (red) and mineral pores (blue).

Fast Field Cycling-NMR

Theoretical expressions of longitudinal spin-relaxation rates for brine and oil diffusing in mineral and kerogen networks

Here, we outline the theoretical relaxation models that consider the longitudinal nuclear relaxation of these two confined fluids. We remind that EMR spectra have evidenced the nature and quantity of the sources of relaxation in both cases. On the mineral lamellar structure, EMR found a density of $\eta_S = 4.5\ 10^{19}\ Mn^{2+}$/g ions in the mineral clay and $\eta_S = 1.2\ 10^{17}\ C^{\circ}$ radicals/g in the kerogen network.

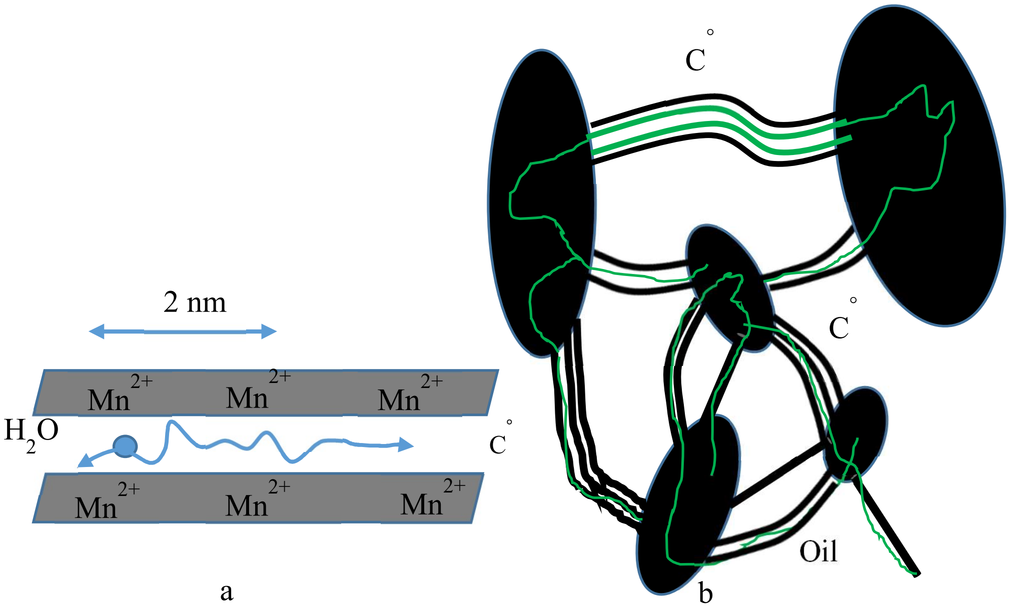

Figure S4: Schematic diagrams representing (a) the brine diffusion within the 2D lamellar clay structure with Mn^{2+} (b) the quasi 1D oil diffusion (green lines) between the micropores of the solid kerogen and larger organic pores in presence of carbon radicals C°.

In the proposed relaxation model, there are basically a liquid and a solid spin densities with longitudinal magnetizations m_{liq} and m_{sol}, respectively. Introducing the dimensionless magnetizations for the departure from equilibrium $M(t)=[m(t)-m^{eq}]/m^{eq}$, these two spin magnetizations are coupled through the following kinetic cross-relaxation equations at the solid-liquid interface:

$$\frac{d}{dt}\begin{pmatrix} M_{sol}(t) \\ M_{liq}(t) \end{pmatrix} = -\begin{pmatrix} R_{1,sol} + k/F & -k/F \\ -k & R_{1,liq} + k \end{pmatrix}\begin{pmatrix} M_{sol}(t) \\ M_{liq}(t) \end{pmatrix}. \qquad (1)$$

Here $R_{1,liq}$ and $R_{1,sol}$ are the longitudinal spin-lattice relaxation rates for the confined liquid and solid-protons in the porous rock, $k\sim 1\ s^{-1}$ is the dipolar cross-relaxation rate from the liquid to the solid proton species and $F = m_{sol}^{eq}/m_{w}^{eq}$ <<1 is the ratio of the solid- and liquid-proton populations at equilibrium (Lester, et al., 1991). The dipolar cross-relaxation rate from the confined liquid to the solid proton species has been chosen as $k\backsim 1s^{-1}$ for a good fit. This value is consistent with an asymptotic limit at low frequency which tends to constant (independent of the frequency) at low frequency. k is thus limited by the transfer of dipolar energy (spin diffusion) within the bound solid proton. The observed plateau below a frequency $\omega_c/2\pi\sim 25$ kHz is thus characteristic of the rigid-lattice limit of the solid proton hydrates R_{sol}.

F<<1 is the ratio of the solid-proton population to the liquid proton at equilibrium (Lester et all 1991), It is thus necessary very small compared to one.

Eq. 1 is a representation of two-component situation with exchange. Even if this approach is seldom used for liquid relaxation in porous solids. This is not the general case, see for instance the case of relaxation in cement-based materials (Barberon, Korb, et al, 2003). If we neglect the exchange, $k \longrightarrow 0$, we find the simple approach of Brownstein and Tarr (Brownstein and Tarr , 1979). However, in that case, it is impossible to find a plateau of $1/T_1 \backsim R_{1,sol}$ at very low frequency and in that case Eqs. 5 or 6 diverge at low frequency. We have thus fitted our NMRD data with the full Eq. 2 as well as Eqs. 5, 6. The values chosen for $R_{1,sol}$ should correspond to $1/T_1(\omega_I<\omega_c\sim 2\pi\ 25\text{kHz})\backsim 500\text{-}600\ s^{-1}$. These values are typical to the numerous hydrates present in the shale samples.

With the fast-field cycling spectrometer that we used; one observes only the signal $M_{liq}(t)$ of the liquid. It is a linear combination of a fast R_{fast}(t) and slow R_{slow}(t) exponential recovery-terms whose amplitudes depend on the

eigenvectors of the matrix in Eq. (1). We show that R_{slow}(t) dominates the relaxation and its analytical expression depends on the different relaxation and cross-relaxation rates through the following relation:

$$R_{1,slow}(\omega_I) = \frac{1}{2}\left\{R_{1,sol} + R_{1,liq}(\omega_I) + k\left(1+\frac{1}{F}\right) - \sqrt{\left[R_{1,sol} - R_{1,liq}(\omega_I) - k\left(1-\frac{1}{F}\right)\right]^2 + \frac{4k^2}{F}}\right\} \quad (2)$$

For porous rocks and at room temperature, the translational diffusion of the embedded liquids is sufficiently fast to ensure the biphasic fast-exchange model between two transient populations of spins:

$$R_{1,liq}(\omega_I) = R_{1,bulk} + \frac{N_S}{N} R_{1,surf}(\omega_I). \quad (3)$$

Here $R_{1,bulk}$ represents the constant bulk spin-lattice relaxation rates of the liquids which come from the relaxation processes induced by rotational and translational molecular dynamics. In Eq. (3), N_S/N represents the surface fraction of the liquid molecules transiently present at pore surfaces:

$$\frac{N_S}{N} = \lambda S_{P,NMR}\rho_{liq}. \quad (4)$$

In Eq. (4), λ is the thickness of a layer of liquid molecules at pore surfaces, $S_{P,NMR}$ and ρ_{liq} are the specific surface area sensed by the NMR technique and the liquid density, respectively. It thus results from Eqs. (3 and 4) that the NMRD frequency dependence of $R_{1,liq}(\omega_I)$ is mainly due to the surface contribution $R_{1,surf}(\omega_I)$.

In the case of water molecules moving transiently at the pore surface in presence of paramagnetic sources of relaxation such Mn^{2+} ions (Figure S4a), we have shown that the frequency dependence of $R_{1,surf}(\omega_I)$ is given by the following bilogarithmic expression (Korb, et al., 2014):

$$R_{1,water}(\omega_I) = R_{1,bulk}^{water} + \frac{\pi}{30\delta_{water}^3}\sigma_S\rho_{water}S_{P,NMR}(\gamma_I\gamma_S\hbar)^2 S(S+1)\tau_m\left\{3ln\left(\frac{1+\omega_I^2\tau_m^2}{(1/A)^2+\omega_I^2\tau_m^2}\right) + 7ln\left(\frac{1+\omega_S^2\tau_m^2}{(1/A)^2+\omega_S^2\tau_m^2}\right)\right\} \quad (5)$$

In Eq. (5), $\lambda_{water} \sim \varepsilon = 0.3\ nm$ where ε is the size of a water molecule is the distance of minimal approach between the moving proton spins (I=1/2) and the immobile electronic spin ($S=5/2$ for Mn^{2+}) at the pore surface. $S_{P,NMR}$ = 47 m^2/g for clays. According to the uniform repartition of paramagnetic species that we found from EMR imaging and considering the respective densities ρ_S[illegible]$2.675\ g/cm^3$ for solid clays, one obtains the surface densities of paramagnetic sources of relaxation $\sigma_S \sim (\eta_S\ \rho_S)^{2/3} = 2.44\ 10^{13}\ Mn^{2+}/cm^2$ is the surface density of Mn^{2+}. τ_m is the surface translational correlation time of the liquid. $A = \tau_S/\tau_m$ represents the dynamical surface affinity or NMR wettability, where [illegible]$_S$ is the time of residence of the liquid molecule at the pore surface (Korb, et al., 2009). Finally, $\omega_I/(2\pi)$ and $\omega_S/(2\pi) = 658\ \omega_I/(2\pi)$ are the Larmor frequencies of the proton and electron spins, respectively.

In the case of oil molecules moving transiently within the complicated sponge-like pore network of kerogen in presence of paramagnetic sources of relaxation such C° radicals and VO^{2+} ions (Figure S4b), we have shown that the frequency dependence of $R_{1,surf}(\omega_I)$ is given by the following expression (Korb, et al., 2014):

$$R_{1,oil}(\omega_I) = R_{1,bulk}^{oil} + \sqrt{2}/\left(15\pi R\delta_{1D,oil}^2\right)\sigma_S\rho_{oil}S_{p,NMR}(\gamma_I\gamma_S\hbar)^2 S(S+1)\sqrt{\tau_m\tau_S}\left[\frac{3\sqrt{1+\sqrt{1+\omega_I^2\tau_S^2}}}{\sqrt{1+\omega_I^2\tau_S^2}} + \frac{7\sqrt{1+\sqrt{1+\omega_S^2\tau_S^2}}}{\sqrt{1+\omega_S^2\tau_S^2}}\right]. \qquad (6)$$

Here $R_{1,bulk}^{oil} \sim 1\ s^{-1}$ has no frequency dependence, $S_{P,NMR} \sim 233\ m^2/g$ for kerogen and the oil density $\rho_{oil} = 0.85\ g/cm^3$, $\sigma_S = 3.26\ 10^{11}$ C°/cm², $\delta_{1D,oil} \sim 0.15\ nm$ and $R \sim 0.3\ nm$ is the dominant radius of the kerogen nanopores power-law distribution within the solid kerogen. The electronic spin in kerogen has been discussed in the paper as being $S=1$ due to the coupling with the VO^{2+} ions

It is shown that below a certain frequency value, ω_c, the system reaches long correlation times typical of the rigid-lattice limit τ_c. k is limited by the transfer of dipolar energy (spin diffusion) within the bound solid-proton. The observed plateau $R_{1,sol}=R_{1,liq}(\omega_I=\omega_c)$ below ω_c is thus characteristic of the rigid-lattice limit of the "solid-protons". Above ω_c, the system experiences correlation times much shorter than τ_c. This favors the condition of weak coupling where $k << R_{1,sol}$ and $R_{1,w}$. The time decay of $M_w(t)$ is now only controlled by the liquid relaxation $R_{1,slow}(\omega_I)=R_{1,liq}(\omega_I)$, where $R_{1,liq}$ is associated to the liquid-proton dynamics in confinement.

One thus obtains the final expressions of $R_{1,slow}(\omega_I)$ by substituting Eqs. (5 or 6) into Eqs. (3 and 2) for a direct comparison with brine or oil NMRD profiles.

Last, Eq. (6) tends to the following asymptotic theoretical temperature and frequency behaviors:

$$T_1 \propto \sqrt{(\omega_I/\tau_m)} = C^{te} \text{ and } T_2(T) \propto 1/\sqrt{(\tau_m\tau_s(T))}. \qquad (7)$$

The invariance of T_1 with the temperature shown in the article is in agreement with the independence of viscosity of a highly confined liquid previously described (Korb, et al., 2015). Similarly, the activated behavior of T_2 with the temperature strongly suggests that the main temperature dependence comes from τ_s and not from τ_m that is inversely proportional to the almost constant surface diffusion coefficient.

The agreement of Eq. 7 with the temperature and frequency dependences of T_1 for oil thus gives a supplementary proof of the validity of our proposed quasi-1D relaxation model.

We show in Figure S5 the observed 2D spin correlation maps T_1-T_2 for oil and brine embedded in shale oils rocks at 2.5 and 23 MHz. Usually in petroleum rocks with large pores, one observes $T_1/T_2 \sim 2$ with a correlation map T_1-T_2 that is only a diagonal. However, in presence of a high confinement with dynamical motion in a 1D or 2D pore system, the ratio T_1/T_2 is drastically enhanced. According to Eqs. 5, 6, one has $T_{1(\omega_I)} \propto \sqrt{\frac{\omega_I}{\tau_m}} = Cte$ whose value depends drastically on the Larmor frequency while $T_2 \propto 1/\sqrt{\tau_m\tau_S}$, has almost no frequency dependence. This is what we

observe in Fig. S5. The ratio T_1/T_2=17 and 74 at 2 and 23 MHz for oil while it is about 5.5 for water. The oil peaks in the 2D T_1-T_2 spectrum moves substantially more than the water peak with increasing frequency. This is consistent with a dynamic of oil on a quasi 1D pore in kerogen compared with a 2D dynamics of water in clay. The comparison between experiments and theory is quite satisfactory and reveals that the oil peak moves substantially more than the water peak with increasing frequency. This result is of particular importance because it shows that measuring T_1-T_2 at different Larmor frequencies gives confidence in characterizing the fluid type present in confinement.

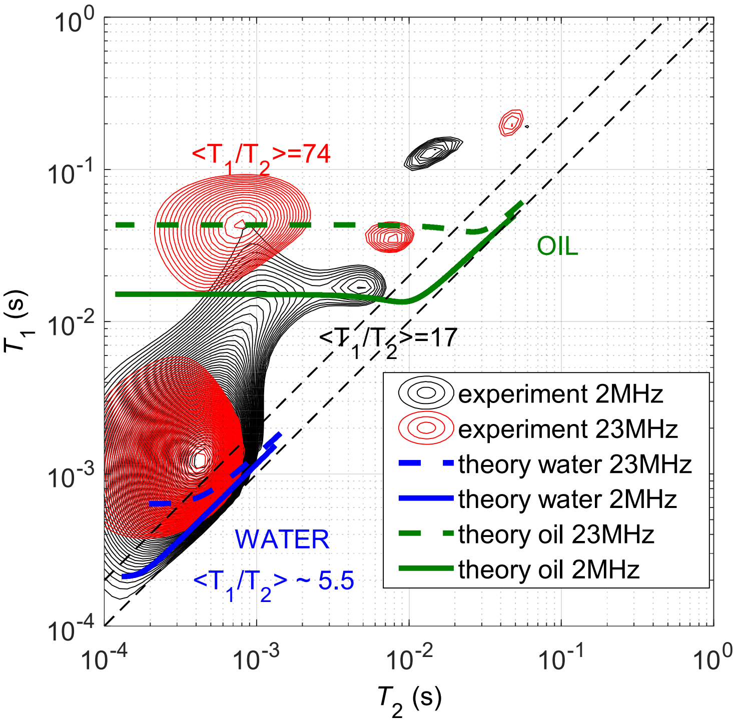

Figure S5: T_1-T_2 correlation maps measured at both 2MHz and 23MHz on an "as received" sample. The lines represent the best fits obtained with the relaxation model described in supplementary material.

EMR Results:

Continuous wave (CW) EMR performed on the dry kerogen isolates show the presence of the carbon radical C^0, but the absence of Mn^{2+} and VO^{2+} signal.

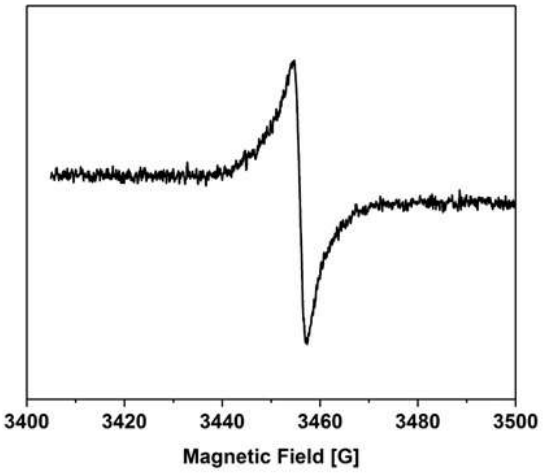

Figure S6 Continuous wave EMR spectrum acquired on dry kerogen isolates showing the presence of carbon radical, but the absence of signal from M_n^{2+} *and* VO^{2+}

We have performed spatial spectral EMR imaging. This technique allows the imaging of a selected signal in the EMR spectrum.

Figure S7 top left shows the imaging obtained on an "as received" sample when all the signal is encoded (both C° and Mn^{2+} transitions). A very similar image is obtained when encoding only the C° transition (Figure S7 top right).

The spectral spatial imaging allows obtaining a full spectrum in each pixel of the image, an example is shown for four different pixels at the bottom of Figure S7. A striking result is that C° and Mn^{2+} transitions are found in every single pixel with different intensities but always with the same ratio. This means that at the resolution of the image (10µm) the carbon radical (located in the organic phase) and the manganese (located in the mineral phase) are always present and with constant proportions.

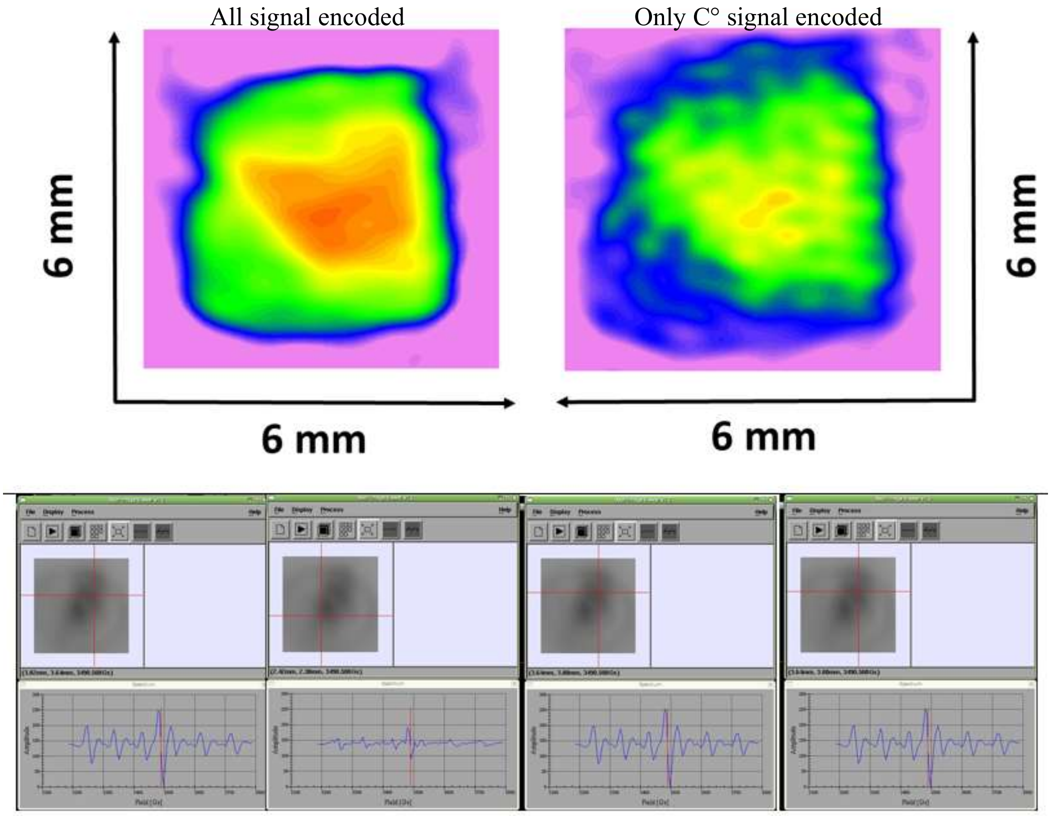

Figure S7 2D spatial imaging of whole EPR spectrum encoded (top left) and kerogen (top right). The image resolution was 10µm per pixel with an image size of 512x512 pixels. The field of view was set to 7 mm and the gradient strength of 175G/ cm. The 2D-spectral spatial imaging (bottom) were recorded with the same conditions using 800 G of spectral width to ensure the detection of the full spectrum. The EMR spectrum were recorded at various spatial positions (bottom) showing a constant ratio between the intensities of central peak (C°) and the six lines (Mn2+).

2D HYSCORE experiments were also conducted in order to confirm the results. On an "as received" sample Figure S8 confirms the absence of proton and carbon coupling on the experiment collected by excitation of the Mn^{2+} transition. This shows that there is no carbon or protons in the close vicinity of the manganese ion. This is an additional information showing that proton and carbon coupling are observed when exciting the C° transition. All these results support the conclusion that carbon radicals are located only in the kerogen phase, while manganese is located only in the mineral phase.

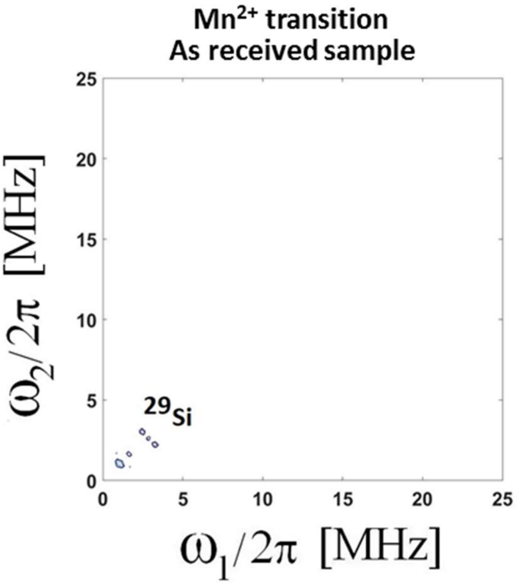

Figure S8 2D HYSCORE experiment collected by excitation of Mn^{2+} (-1/2) transition on "as received" sample showing the absence of carbon or proton features

We showed in the article the appearance of proton and carbon feature on the 2D HYSCORE experiment of carbon centered radicals when dodecane was imbibed in kerogen. In Figure S9 we show that after removal of the dodecane, the carbon and proton feature disappear, proving the reversibility of the process.

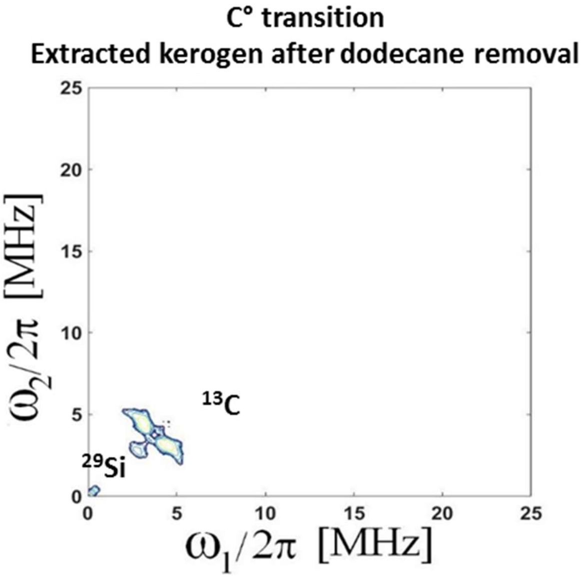

Figure S9 2D HYSCORE collected by excitation of C° transition on extracted kerogen after dodecane removal showing the reversibility of the magnetic locking

In order to verify the presence of vanadyl porphyrin, a 2D HYSCORE was performed by exciting the VO^{2+} (-3/2) transition. Figure S10 shows the typical ^{14}N pattern of a porphyrin structure (Gourier, et al., 2010).

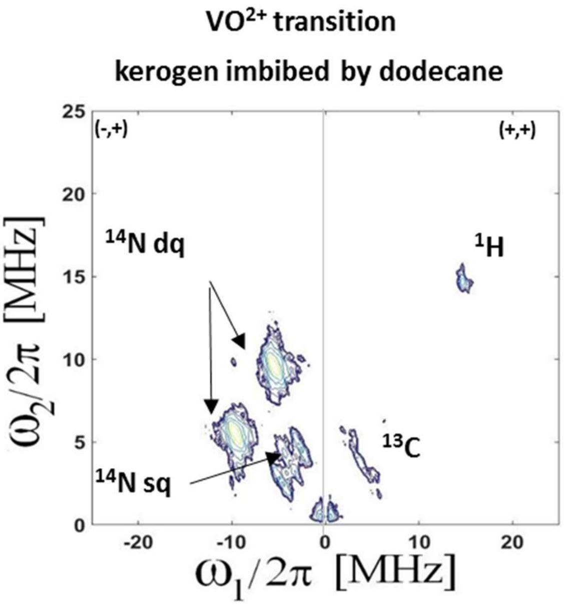

Figure S10 2D HYSCORE collected by excitation of VO^{2+} (-3/2) transition on kerogen imbibed by dodecane showing the typical signal of vanadyl porphyrin.^{14}N double quanta (dq) and single quantum (sq) are shown.

Références

Barberon, F; Korb, J.-P. [et al] Probing the surface area of a cement-based material by nuclear magnetic relaxation dispersion [Article] Phys rev. Lett. 90 2003, 116103.

Ben Tayeb K. [et al.] Applications of Pulsed Electron Paramagnetic Resonance Spectroscopy to the Identification of Vanadyl Complexes in Asphaltene Molecules. Part 1: Influence of the Origin of the Feed [Article] // Energy Fuel. - 2015. - p. 4608−4615.

Ben Tayeb K. [et al.] Applications of Pulsed Electron Paramagnetic Resonance Spectroscopy to the Identification of Vanadyl Complexes in Asphaltene Molecules. Part 2: Hydrotreatment Monitoring [Article] // Energy & Fuels. - 2017. - pp. 3288–3294.

Breit G.N. and Wanty R.B. Vanadium accumulation in carbonaceous rocks: A review of geochemical controls during deposition and diagenesis [Journal] // Chemical Geology. - 1991. - 2 : Vol. 91. - pp. 83-97.

Curtis M. E., Ambrose R. J. and Sondergeld C. H. Structural Characterization of Gas Shales on the Micro- and Nano-Scales [Journal] // SPE 137693. - 2010.

Derenne S. [et al.] Molecular evidence for life in the 3.5 billion year old warrawoona chert [Journal] // Earth Planet Science Letters. - 2008. - pp. 476-480.

Ertas D., Kelemen S.R. and Halsey T.C. Petroleum Expulsion Part 1. Theory of Kerogen Swelling in [Journal] // Energy & Fuels. - 2006. - pp. 295-300.

Fialips C.I. [et al.] Quantitative mineralogy of Vaca Muerta and Alum Shales from core chips and drill cuttings by calibrated SEM-EDS mineralogical mapping [Conference] // Unconventional Resources Technology Conference. - Houston, Texas, USA : [s.n.], 2018.

Gourier D. [et al.] Extreme deuterium enrichment of organic radicals in the Orgueil meteorite: Revisiting the interstellar interpretation? [Article] // Geochimica et Cosmochimica Acta. - 2008. - pp. 1914–1923.

Gourier Denis [et al.] EPR, ENDOR, and HYSCORE study of the structure and the stability of vanadyl-porphyrin complexes encapsulated in silica: Potential paramagnetic biomarkers for the origin of life [Article] // Journal of Physical Chemistry B. - 2010. - pp. 3714–3725.

Handwerger D. A., Keller J. and Vaughn K. Improved Petrophysical Core Measurements on Tight Shale Reservoirs Using Retort and Crushed Samples [Journal] // SPE 147456. - 2011.

Handwerger D.A. [et al.] Reconciling Retort versus Dean Stark Measurements on Tight Shales [Conference] // SPE 159976. - 2012.

Hofer P. Distortion-Free Electron-Spin-Echo Envelope-Modulation Spectra of Disordered Solids Obtained from Two-Dimensional and Three-Dimensional HYSCORE Experiments [Journal] // Journal of Magnetic Resonance. - 1994. - 1 : Vol. 111. - pp. 77-86.

Jeschke G. [et al.] DeerAnalysis2006—a comprehensive software package for analyzing pulsed ELDOR data [Journal] // Applied Magnetic Resonance. - 2006. - 3-4 : Vol. 30. - pp. 473-498.

Jeschke G. [et al.] Direct conversion of EPR dipolar time evolution data to distance distributions [Journal] // Journal of Magnetic Resonance. - 2002. - Vol. 033. - pp. 72-82.

Kimmich R. NMR Tomography, Diffusometry, Relaxometry [Book]. - [s.l.] : Springer, 1997.

Korb J.P. [et al.] Dynamical surface affinity of diphasic liquids as a probe of wettability of multimodal porous media [Journal] // Phys. Rev. E. - 2009. - 6 : Vol. 80. - pp. 1601-1612.

Korb J.P. [et al.] Dynamics and Wettability of Oil and Water in Oil Shales [Journal] // Journal of Physical Chemsitry C. - 2014. - Vol. 118. - pp. 23212-23218.

Korb J.P. [et al.] Relation and correlation between NMR relaxation times, diffusion coefficients, and viscosity of heavy crude oil [Journal] // J. Phys. Chem. C. - 2015. - 43 : Vol. 119. - pp. 24439-24446 .

Korb J.P., Nicot B. and Jolivet I. Dynamics and wettability of petroleum fluids in shale oil probed by 2D T1-T2 and fast field cycling NMR relaxation [Journal] // Microporous and Mesoporous Materials. - 2018. - Vol. 269. - pp. 7-11.

Le Bihan A. [et al.] Quality Control of Porosity and Saturation Measurements on Source Rocks [Journal] // SPWLA. - 2014.

Lee T., Bocquet L. and Coasne B. Activated desorption at heterogeneous interfaces and long-time kinetics of hydrocarbon recovery from nanoporous media [Journal] // Nature Communications. - 2016.

Lester C.C. and Bryant R.G. Water-proton nuclear magnetic relaxation in heterogeneous systems: hydrated lysozyme results [Journal] // Magnetic Resonance in Medecine. - 1991. - 1 : Vol. 22. - pp. 143-153.

Loucks R. [et al.] Spectrum of pore types and networks in mudrocks and a descriptive classification for matrix related pores [Journal] // AAPG Bulletin. - 2012. - pp. 1071-1098.

Mills R. and Lobo V.M.M. self-diffusion in electrolyte solutions, a critical examination of data compiled from literature [Book]. - Amsterdam : Elsevier, 1989.

Nicot B. [et al.] Estimating Saturations in Organic Shales using 2D NMR [Journal] // SCA 24. - 2015.

Simpson G.A. and N.S. Fishman Unconventional Tight Oil Reservoirs: A Call for New Standardized Core Analysis Workflows and Research [Journal] // SCA 22. - 2015.

Singer P.M., Chen Z. and Hirasaki G. Fluid Typing and Pore Size in Organic Shale Using 2D NMR in Saturated Kerogen Isolates [Journal] // Petrophysics. - 2016.

Sondergeld C.H. [et al.] Petrophysical Considerations in Evaluating and Producing Shale Gas Resources [Journal] // SPE 131768. - 2010.

Spellman F.R. Environmental Impacts of Hydraulic Fracturing [Book]. - [s.l.] : CRC Press, 2013.

Tomassini F.G. [et al.] 'Co-opetition': a game-changing synergy among operators. A case delivering a Rosetta Stone for the Vaca Muerta Stratigraphy (Argentina) [Conference] // Unconventional Resources Technology Conference. - 2016.

Venkataramanan L. Solving Fredholm Integrals of the First Kind With Tensor Product Structure in 2 and 2.5 Dimensions [Journal] // IEEE TRANSACTIONS ON SIGNAL PROCESSING. - 2002. - pp. 1017-1026.
